# CREB Negatively Regulates *IGF2R* Gene Expression and Downstream Pathways to Inhibit Hypoxia-Induced H9c2 Cardiomyoblast Cell Death

**DOI:** 10.3390/ijms161126067

**Published:** 2015-11-24

**Authors:** Wei-Kung Chen, Wei-Wen Kuo, Dennis Jine-Yuan Hsieh, Hsin-Nung Chang, Pei-Ying Pai, Kuan-Ho Lin, Lung-Fa Pan, Tsung-Jung Ho, Vijaya Padma Viswanadha, Chih-Yang Huang

**Affiliations:** 1 Department of Emergency Medicine, China Medical University Hospital, Taichung 40402, Taiwan; ercwk@mail.cmuh.org.tw (W.-K.C.); linalpra@hotmail.com (K.-H.L.); 2 Department of Biological Science and Technology, China Medical University, Taichung 40402, Taiwan; wwkuo@mail.cmu.edu.tw; 3 School of Medical Laboratory and Biotechnology, Chung Shan Medical University, Taichung 40201, Taiwan; djh@csmu.edu.tw; 4 Graduate Institute of Clinical Medical Science, China Medical University, Taichung 40402, Taiwan; dadalidada0809@gmail.com; 5 Division of Cardiology, China Medical University Hospital, Taichung 40402, Taiwan; paidoctor1@gmail.com; 6 College of Medicine, China Medical University, Taichung 40402, Taiwan; 7 Cardiology Department, Taichung Armed Forces General Hospital. Taichung 41152, Taiwan; lung-fa@803.org.tw; 8 Department of Medical Imaging and Radiological Sciences, Central Taiwan University of Science and Technology, Taichung 40601, Taiwan; 9 School of Chinese Medicine, College of Chinese Medicine, China Medical University, Taichung 40402, Taiwan; tjho@mail.cmu.edu.tw; 10 Chinese Medicine Department, China Medical University Beigang Hospital, Yunlin 651, Taiwan; 11 Department of Biotechnology, Bharathiar University, Coimbatore-641 046, India; padma.vijaya@gmail.com; 12 Graduate Institute of Basic Medical Science, China Medical University, Taichung 40402, Taiwan; 13 Department of Health and Nutrition Biotechnology, Asia University, Taichung 41354, Taiwan

**Keywords:** CREB, hypoxia, apoptosis, IGF2R signaling

## Abstract

During hypoxia, gene expression is altered by various transcription factors. Insulin-like growth factor-II (IGF2) is known to be induced by hypoxia, which binds to IGF2 receptor IGF2R that acts like a G protein-coupled receptor, might cause pathological hypertrophy or activation of the mitochondria-mediated apoptosis pathway. Cyclic adenosine monophosphate (cAMP) responsive element-binding protein (CREB) is central to second messenger-regulated transcription and plays a critical role in the cardiomyocyte survival pathway. In this study, we found that IGF2R level was enhanced in H9c2 cardiomyoblasts exposed to hypoxia in a time-dependent manner but was down-regulated by CREB expression. The over-expression of CREB in H9c2 cardiomyoblasts suppressed the induction of hypoxia-induced IGF2R expression levels and reduced cell apoptosis. Gel shift assay results further indicated that CREB binds to the promoter sequence of *IGF2R*. With a luciferase assay method, we further observed that CREB represses *IGF2R* promoter activity. These results suggest that CREB plays an important role in the inhibition of IGF2R expression by binding to the *IGF2R* promoter and further suppresses H9c2 cardiomyoblast cell apoptosis induced by IGF2R signaling under hypoxic conditions.

## 1. Introduction

The transcription factor cAMP responsive element-binding protein (CREB) is a 43 kDa transcription factor that specifically binds DNA at the cyclic adenosine monophosphate (cAMP) responsive element-binding protein (CREB) within the regulatory regions of CREB target genes [1]. CREB acts as a downstream effector in many signaling pathways and is capable of being activated by numerous pathways including cAMP/protein kinase A-mediated signaling, mitogen-activated protein kinase (MAPK)/extracellular signal-regulated kinases(ERK), or the Ca^2+^/calmodulin-dependent protein kinase II/IV (Ca^2+^/CaMKII/IV) pathway. These pathways are mainly regulated in response to growth factors, stress signals, intracellular Ca^2+^, and specific peptides [2,3,4]. CREB can be phosphorylated at Ser-133 by variety of kinases, including protein kinase A (PKA), Akt, and ribosome S6 kinase (p90^RSK2^) to increase CREB-dependent gene transcription [5,6,7]. Phosphorylation of CREB is essential for binding to the DNA CRE site via a leucine zipper domain, which then leads to the recruitment of several co-activator proteins to assist with transcription [3,8,9].

CREB is a downstream component of the insulin/insulin-like growth factor (IGF) cascade, which plays crucial roles in maintaining cell viability and embryo survival [2] and has also been reported to be required for cell survival in neurons and pancreatic cells and the control of glucose and lipid metabolism in hepatic cells [10,11,12,13]. CREB is known to be involved in the maintenance of normal ventricular structure and function of the heart [14]. The anti-apoptotic effects of IGF-I require both PI3K- and MEK1-involved signaling mechanisms, in order to activate CREB in cardiomyocytes, which further induces the anti-apoptotic bcl-2 expression [15]. 

Apoptosis has been identified to play a role in many cardiovascular diseases, including myocardial infarction and heart failure, due to cardiomyocyte loss and dysfunction [16,17]. New evidence has shown that cells under stress such as hypoxia secrete Insulin-like growth factor-II (IGF2) and promote IGF2 receptor (IGF2R) expression [18,19]. IGF2R has been identified as a death receptor gene involved in apoptosis [20]. In cardiomyoblast cells, IGF2R plays a critical role in the regulation of cell apoptosis, which might contribute to heart failure [21]. IGF2 binds to IGF2R, that acts like a G protein-coupled receptor, and involves in promoting pathological hypertrophy via Gq protein (Gαq) and its downstream effectors such as protein kinase C (PKC)-α, CaMKII, and calcineurin to induce the expression of fetal gene atrial natriuretic peptide (ANP) and brain natriuretic peptide (BNP) [19,21]. Gαq, via phosphorylation of phospholipase C-δ3 (PLC-δ3) on Ser-537, contributes to the triggering of calcium influx and pathways involving PKC-α, calcineurin, and CaMKII [21,22].

Previous studies have shown that gene expression during hypoxia is regulated by specific transcription factors, such as CREB and NF-κB [15,23]. In cardiomyocytes, hypoxia is associated with phosphorylation of CREB, which is mediated by IGF-I and involve PI3K- and MEK1-activation, which then induces bcl-2 [15]. 

However, the molecular mechanisms underlying the role of exogenous or endogenous CREB in the regulation of hypoxia-induced IGF2R and cell apoptosis in the heart remain poorly understood. We proposed that hypoxic conditions would stimulate IGF2R activation, thereby triggering downstream signaling cascades, promoting cardiomyocyte apoptosis and, finally, resulting in heart failure. CREB potentially plays a crucial role in providing protection from hypoxia-induced IGF2R signaling and apoptosis in H9c2 cardiomyoblast cells, thereby it inhibits heart failure. In this study, we report that CREB plays a critical role in negatively regulating *IGF2R* gene expression in hypoxia. CREB, as a transcriptional repressor, binds to the *IGF2R* promoter region and further protects against hypoxia-induced heart disease.

## 2. Results

### 2.1. cAMP Responsive Element-Binding Protein (CREB) Was Involved in Cell Survival in H9c2 Cardiomyoblast Cells

**Figure 1:**
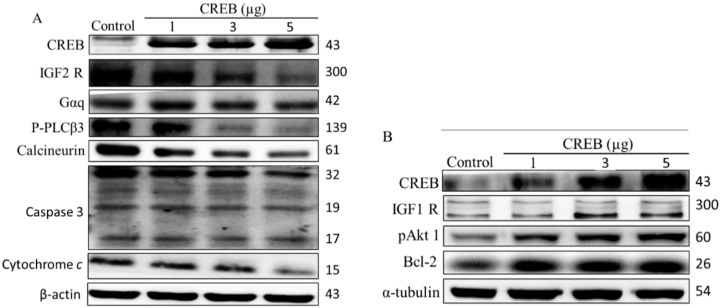
Cyclic adenosine monophosphate (cAMP) responsive element-binding protein (CREB) is involved in the regulatory IGF1R and IGF2R cell signaling pathways in H9c2 cells. To assess the role of CREB in IGF1R and IGF2R signaling pathways, protein levels under normoxia were measured by Western blotting (**A**,**B**). H9c2 cardiomyoblast cells were transiently transfected with 1–5 μg of CREB plasmid for 24 h.

Previous studies have shown that the anti-apoptotic effect of IGF-I require activation of the transcription factor CREB and subsequent expression of bcl-2 [15]. Therefore, we further characterized the IGF1R and IGF2R pathway-related protein expression in H9c2 cardiomyoblast cells after 24 h of CREB transfection (Figure 1A,B). The levels of IGF2R pathway-related IGF2R, Gαq, p-PLCβ3, calcineurin, caspase-3, and cytochrome-C protein expression were significantly decreased in H9c2 cardiomyoblast cells after transfection with CREB in a dose-dependent manner (Figure 1A). The expression of IGF1R pathway-related proteins IGF1R, p-AKT, and Bcl-2 increased with the transfection of increasing doses of CREB (Figure 1B). These findings suggest that CREB overexpression not only repressed the IGF2R-induced apoptosis pathway but also improved cell survival by activating the anti-apoptotic factor Bcl-2 in H9c2 cardiomyoblast cells.

### 2.2. Hypoxia-Induced Apoptosis Was Suppressed by CREB Transfection in H9c2 Cardiomyoblasts

**Figure 2:**
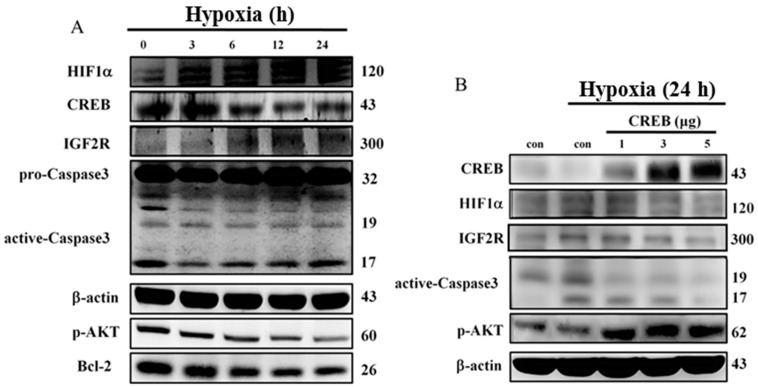
Hypoxia-induced apoptosis was blocked by transfection with CREB in H9c2 cells. (**A**) The effects of hypoxia on the protein levels of CREB, HIF1α, IGF2R, caspase-3, p-AKT and Bcl-2, as detected by Western blot; and (**B**) CREB over-expression further reduced HIF1α, IGF2R, and caspase-3 protein expression levels to inhibit hypoxia-induced apoptosis in H9c2 cells. After being transfected with CREB plasmid for 24 h, H9c2 cells were treated with hypoxia for 24 h. Hypoxia-induced apoptosis-related proteins were measured by Western blot.

To investigate the molecular mechanisms that regulate the hypoxia-induced anti-apoptotic effect of CREB, we first confirmed that cell damage was induced by hypoxia. After 24 h of hypoxia exposure, the expression levels of hypoxia-induced factors, cell apoptosis proteins, and cell survival proteins in H9c2 cardiomyoblast cell were detected by Western blotting. The protein levels of HIF1α, IGF2R, p-IGF2R, and caspase-3 were elevated in a time-dependent manner, but the levels of CREB, p-AKT, and bcl-2 were reduced when the cells were exposed to hypoxia (Figure 2A). These data imply that CREB expression is repressed by hypoxia and that hypoxia induces mitochondrial-dependent cell apoptosis. Further transfection with CREB plasmids resulted in a dose-depended reversion in the hypoxia-induced reduction of p-AKT signaling in H9c2 cardiomyoblasts. In addition, the increased expression of the apoptosis proteins HIF1α, IGF2R, and caspase-3 proteins induced by hypoxia was attenuated following transfection with different doses of CREB (Figure 2B).

### 2.3. CREB Translocated to the Nuclei of H9c2 Cells to Directly Target the Promoter Region of the IGF2R

**Figure 3:**
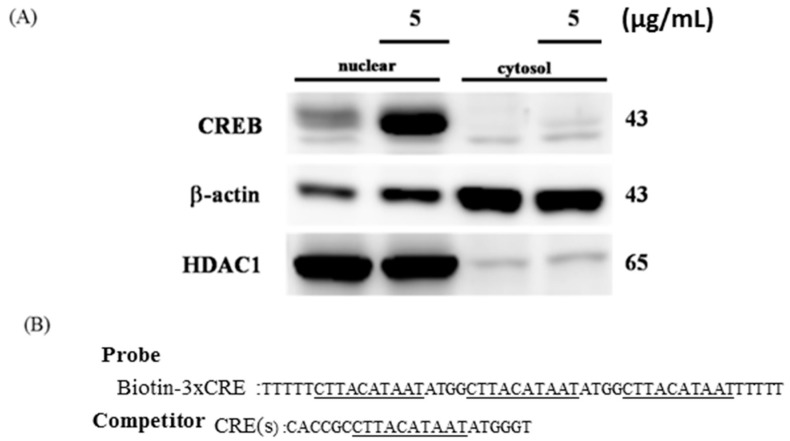

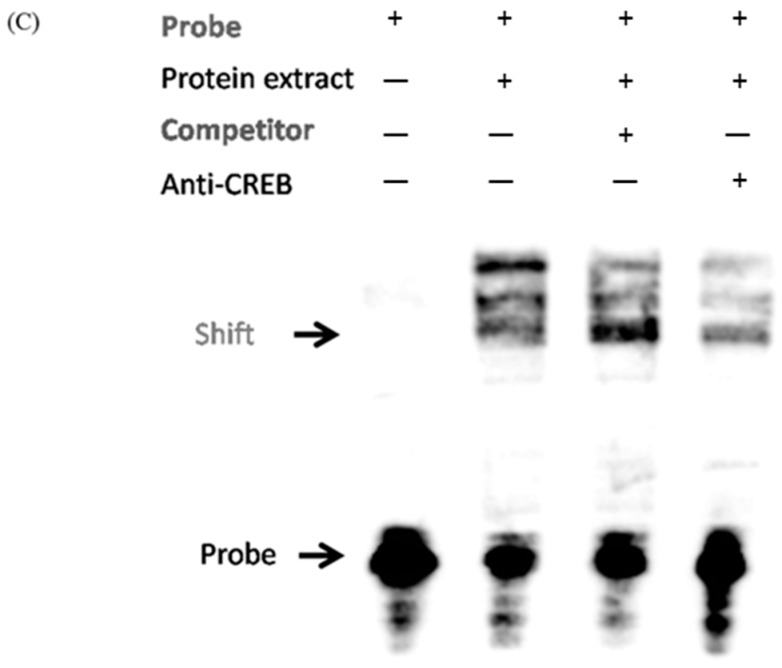
CREB translocated to the nucleus of H9c2 cells and directly targeted the promoter region of IGF2R. (**A**) Nuclear protein extracts were prepared from H9c2 cells transfected with or without CREB; (**B**) the sequences of oligonucleotides used for the gel-shift, the EBPRE consensus sequences are marked by underlines.; and (**C**) the EMSA pattern showing appropriate band shift in the presence (+) of probe and in the absence (—) of either the competitor or anti-CREB. EMSA was performed in triplicate.

To confirm that nuclear translocation of CREB occurs in H9c2 cells, nuclear, and cytoplasmic extractions were performed after transfecting H9c2 cells with the CREB plasmid for 24 h. The results indicated that CREB translocates to the nucleus in H9c2 cells (Figure 3A). To identify whether CREB directly binds to the EBPRE sequence, an electrophoretic mobility-shift assay (EMSA) was carried out with biotin-labeled double-stranded DNA (Figure 3B). CREB, when over expressed in H9c2 cardiomyoblasts, bound to the probe with the three repeats of the EBPRE sequence. The band shift was attenuated on addition of either biotin-free double-stranded EBPRE DNA sequence or an anti-CREB antibody (Figure 3C).

### 2.4. CREB Suppresses IGF2R Expression by Negatively Regulating the IGF2R Promoter Activity

**Figure 4:**
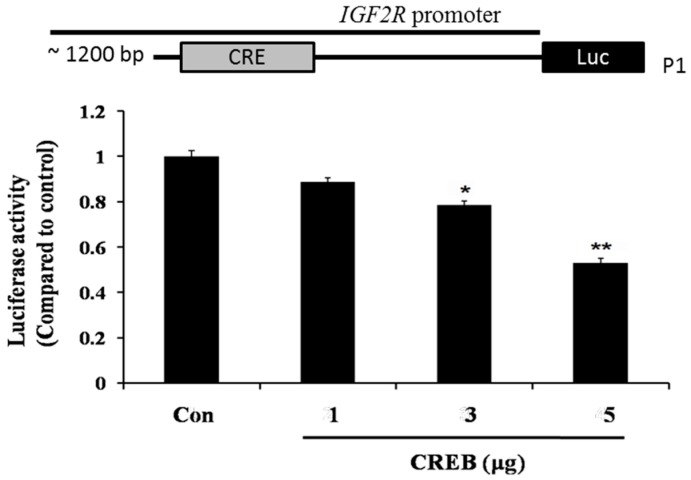
CREB suppressed IGF2R gene expression by directly targeting the promoter region of IGF2R. Schematic diagrams of the *IGF2R* gene indicating predicted EBPRE sites. H9c2 cells were transiently co-transfected with empty or CREB over-expression vectors and with luciferase-reporter constructs (P1) containing ~1.2 kb of the *IGF2R* promoter, either containing or not containing the predicted EBPRE site. Luciferase activity is compared with the control. * *p* < 0.05, ** *p* < 0.001 represent significance when compared to control.

A construct containing −890 to −899 EBPRE-containing region of the *IGF2R* promoter with luciferase reporter was used to monitor *IGF2R* gene transcription. The results showed that CREB caused a significant reduction in *IGF2R* promoter activity when compared with control (a vehicle containing empty plasmid) in a dose-dependent fashion (Figure 4). Taken together, these data suggest that CREB directly binds to the EBPRE sequence of *IGF2R* promoter and repress *IGF2R* gene transcription in H9c2 cardiomyoblasts.

## 3. Discussion

Various mechanisms govern cardioprotection against various stresses. Mild hypoxia *in vitro* is known to be cytoprotective in H9c2 cells [24]. It has also been reported that different degrees of hypoxia exert cardioprotection via activating different signaling pathways involving either ERK1/2 or Akt or ATP [25,26,27]. However the mechanism of cardioprotection established against the effects of hypoxia is not well established. Our previous study indicates that while short term intermittent hypoxia induced protective effects on hearts, long-term intermittent hypobaric hypoxia caused deleterious effects. Therefore deleterious or advantageous effect of cardiac adaptation against intermittent hypobaric hypoxia is highly dependent on the time–course of exposure [28,29,30].

In this study, we investigated the role of CREB in the regulatory mechanisms behind hypoxia-induced, IGF2R-dependent cell apoptosis in H9c2 cells. CREB is a ubiquitously expressed transcription factor that plays an important role in regulating cellular differentiation and proliferation and participates in the development of cancer and atherosclerosis [31,32]. CREB has been shown to bind to specific DNA sequences, TGACGTAA, termed cAMP-response elements (CREs) involved in controlling protein expressions associated with cellular proliferation and apoptosis [33], and the transcriptional activity of these genes is regulated by numerous kinases such as PKA and GSK-3 [34,35]. 

Recent studies have reported that CREB is a potent inhibitor of the ER stress-signaling cascade [36], and attenuation of CREB activation promotes apoptosis [37], CREB has been also known to suppress okadaic acid-induced apoptosis in PC12 cells [38]. Our findings demonstrated that the over-expression of CREB decreased IGF2R protein levels in a dose-dependent manner, suggesting that CREB may be involved in H9c2 cell survival (Figure 1).

IGF2R, a ~300 kDa cation-independent mannose 6-phosphate (M6P) receptor, is a multifunctional protein that interacts with diverse intracellular and extracellular ligands and drives cells toward apoptosis [39,40]. Previous studies have demonstrated that IGF2R activates the mitochondria-mediated apoptotic pathway via Gαq, a GTP-binding protein, and further activates the calcium (Ca^2+^)-calcineurin-mediated dephosphorylation of Bad [21,41,42]. Our findings also demonstrate that hypoxia enhances IGF2R level in H9c2 cells (Figure 2A). However, the IGF2R expression and the cellular apoptosis induced by hypoxia were attenuated by CREB (Figure 2B). Our data further reveal that CREB translocates to the nucleus and directly binds to the CRE site of the *IGF2R* promoter (Figure 3C). Additionally, the luciferase assay showed that the binding of CREB to the *IGF2R* promoter suppresses *IGF2R* gene expression (Figure 4). 

CREB is a member of the mammalian basic leucine zipper (bZIP) transcription factor superfamily [7,43]. Other members of this superfamily of transcription factors include the nuclear factor IL-3 (NFIL3), activator protein 1, CCAAT enhancer binding protein (C/EBP), nuclear factor (erythroid-derived 2), and proline and acidic residue rich (PAR) families. Previous studies have shown that the NFIL3 consensus sequence EBPRE is similar to the cAMP response element. In addition, NFIL3 and CREB compete for the same binding sites and control gene transcription together [44,45]. Our previous study reveals that NFIL3 suppresses hypoxia-induced apoptosis by binding to the promoter of *IGF2R* [46]. Recognition of the same *cis*-binding element by NFIL3, CREB, and C/EBPβ has been known to spur interesting interactions of these transcription factors to regulate identical target gene expression. In addition, NFIL3 is known to heterodimerize with CREB and inhibit DNA binding [47]. 

Our results revealed that IGF2R protein expression was induced under hypoxic conditions and that H9c2 cell apoptosis was negatively regulated by CREB acting on the CRE site. This finding provides new insight into the actions of CREB against hypoxia-induced heart diseases via the suppression of *IGF2R* gene transcription and further inhibition of cardiomyoblast apoptosis.

## 4. Materials and Methods

### 4.1. Cell Culture and Transfection

H9c2 cells were obtained from American Type Culture Collection (ATCC) and were cultured in Dulbecco’s modified essential medium supplemented with 10% fetal bovine serum, 2 mM glutamine, 100 U/mL penicillin, 100 mg/mL streptomycin, and 1 mM pyruvate in humidified air (5% CO_2_) at 37 °C. For hypoxia experiments, the cells were pre-incubated in minimal essential medium for 24 h and subsequently the H9c2 cells were cultured with 5% CO_2_, 94% N_2_, and 1% O_2_ at 37 °C. Cell-based assays were carried out with cell density of up to 70%. CREB plasmids were procured from OriGene (Level Biotechnology Inc., Taipei, Taiwan), and H9c2 cells were transfected for using PureFection reagents (System Biosciences, Mountain View, CA, USA) for 24 h.

### 4.2. Western Blot

For Western blot analysis, H9c2 cells were washed with PBS and lysed in lysis buffer (50 mM Tris, pH 7.5; 0.5 M NaCl; 1.0 mM EDTA, pH 7.5; 10% glycerol; 1 mM basal medium Eagle; 1% Igepal-630; proteinase inhibitor cocktail tablet (Roche, Mannheim, Germany)) and the debris were pelleted by centrifuging at 12,000× *g* for 30 min. The supernatants were electrophoresed by sodium dodecyl sulfate-Polyacrylamide gel electrophoresis (SDS–PAGE) and transferred onto Immobilon™ PVDF membranes (Millipore, Bedford, MA, USA). The membranes were blocked using 5% low-fat milk and 1% Tween 20 in PBS and the blotted proteins were probed with one of the following antibodies: anti-NFIL3, anti-Bax, anti-Bak, anti-Cyt C, anti-cleaved caspase-3, anti-α-tubulin, anti-β-actin (Santa Cruz Biotechnology, Santa Cruz, CA, USA), anti-IGF2, anti-IGF2R (Abcam, Cambridge, MA, USA), anti-IGF1R, anti-phospho-IGF1R, anti-PI3K, anti-phospho-Akt, and anti-phospho-PLCβ (Cell Signaling Technology, Beverly, MA, USA). Protein expression was detected with the ECL detection system (Millipore). 

### 4.3. Nuclear Extracts and the Electrophoretic Mobility Shift Assay (EMSA)

H9c2 cell nuclear extract was prepared using Nuclear and Cytoplasmic Extraction Reagents (Thermo, Grand Island, NY, USA) following the manufacturers instruction as reported earlier [46]. Briefly, nuclear extracts, binding mixtures, the competitor and the anti-NFIL3 antibody (Santa Cruz) were incubated on ice for 5 min. The labeled probe was then added and incubated for an additional 25 min. The reaction products were run by electrophoresis in a 6% non-denaturing polyacrylamide gel and were then transferred to Nylon Membrane (Thermo). The products were viewed using chemiluminescence (Thermo). The sequence of the probes used was: 5′-TTTTTCTTACATAATATGGCTTACATAATATGGCTTACATAATTTTTT-3′ and that of the competitor was: 5′-CACCGCCTTACATAATATGGGT-3′.

### 4.4. Luciferase Assay

The luciferase assay was done as mentioned in our previous reports [46]. IH9c2 cells plated in 6-well plates at 50%–70% confluence were transfected with either pCMV-CREB construct or an equal amount of empty pCMV vector and either the pGL4-IGF2R_P1 construct utilizing PureFection™ reagents (System Biosciences). The cells were incubated in 10% DMEM for 24 h and the luciferase assay was performed using the Dual-Luciferase Reporter Assay System (Promega, Tokyo, Japan). The assay was performed in triplicates. GF2R-luciferase constructs were created by inserting ~1.3 and ~0.7 kb fragments of the predicted EBPRE sequence into the pGL4-BASIC-luciferase plasmid (Promega). The primers used to amplify the DNA fragments were as follows: IGF2R_P1 (forward: 5′-TCTCATCTCGAGCAATGACTAGTCTTCATGTAACAGC-3′ and reverse: 5′-GCCGC-AAAGCTTGAGTCGAAGCTGCAACGG-3′) and A commercial plasmid containing a CMV-driven Renilla reporter system was used as an internal control (Promega).

## References

[1] Hoeffler J.P., Meyer T.E., Yun Y., Jameson J.L., Habener J.F. (1988). Cyclic AMP-responsive DNA-binding protein: Structure based on a cloned placental cDNA. Science.

[2] Schindler M., Fischer S., Thieme R., Fischer B., Santos A.N. (2013). cAMP-responsive element binding protein: A vital link in embryonic hormonal adaptation. Endocrinology.

[3] Hansen R.T., Zhang H.T. (2013). Senescent-induced dysregulation of cAMP/CREB signaling and correlations with cognitive decline. Brain Res..

[4] Davis S., Vanhoutte P., Pages C., Caboche J., Laroche S. (2000). The MAPK/ERK cascade targets both Elk-1 and cAMP response element-binding protein to control long-term potentiation-dependent gene expression in the dentate gyrus *in vivo*. J. Neurosci..

[5] Lamph W.W., Dwarki V.J., Ofir R., Montminy M., Verma I.M. (1990). Negative and positive regulation by transcription factor cAMP response element-binding protein is modulated by phosphorylation. Proc. Natl. Acad. Sci. USA.

[6] Ofir R., Dwarki V.J., Rashid D., Verma I.M. (1991). CREB represses transcription of fos promoter: Role of phosphorylation. Gene Expr..

[7] Mayr B., Montminy M. (2001). Transcriptional regulation by the phosphorylation-dependent factor CREB. Nat. Rev. Mol. Cell Biol..

[8] Meyer T.E., Habener J.F. (1993). Cyclic adenosine 3′,5′-monophosphate response element binding protein (CREB) and related transcription-activating deoxyribonucleic acid-binding proteins. Endocr. Rev..

[9] Lee K.A., Masson N. (1993). Transcriptional regulation by CREB and its relatives. Biochim. Biophys. Acta.

[10] Lonze B.E., Ginty D.D. (2002). Function and regulation of CREB family transcription factors in the nervous system. Neuron.

[11] Liu W., Chin-Chance C., Lee E.J., Lowe W.L. (2002). Activation of phosphatidylinositol 3-kinase contributes to insulin-like growth factor I-mediated inhibition of pancreatic β-cell death. Endocrinology.

[12] Herzig S., Hedrick S., Morantte I., Koo S.H., Galimi F., Montminy M. (2003). CREB controls hepatic lipid metabolism through nuclear hormone receptor PPAR-γ. Nature.

[13] Herzig S., Long F., Jhala U.S., Hedrick S., Quinn R., Bauer A., Rudolph D., Schutz G., Yoon C., Puigserver P. (2001). CREB regulates hepatic gluconeogenesis through the coactivator PGC-1. Nature.

[14] Fentzke R.C., Korcarz C.E., Lang R.M., Lin H., Leiden J.M. (1998). Dilated cardiomyopathy in transgenic mice expressing a dominant-negative CREB transcription factor in the heart. J. Clin. Investig..

[15] Mehrhof F.B., Muller F.U., Bergmann M.W., Li P., Wang Y., Schmitz W., Dietz R., von Harsdorf R. (2001). In cardiomyocyte hypoxia, insulin-like growth factor-I-induced antiapoptotic signaling requires phosphatidylinositol-3-OH-kinase-dependent and mitogen-activated protein kinase-dependent activation of the transcription factor cAMP response element-binding protein. Circulation.

[16] Kang P.M., Izumo S. (2000). Apoptosis and heart failure: A critical review of the literature. Circ. Res..

[17] Huang C.-Y., Lee S.-D. (2012). Possible pathophysiology of heart failure in obesity: Cardiac apoptosis. BioMedicine.

[18] Chu C.H., Lo J.F., Hu W.S., Lu R.B., Chang M.H., Tsai F.J., Tsai C.H., Weng Y.S., Tzang B.S., Huang C.Y. (2012). Histone acetylation is essential for ANG-II-induced IGF-IIR gene expression in H9c2 cardiomyoblast cells and pathologically hypertensive rat heart. J. Cell. Physiol..

[19] Chu C.H., Tzang B.S., Chen L.M., Kuo C.H., Cheng Y.C., Chen L.Y., Tsai F.J., Tsai C.H., Kuo W.W., Huang C.Y. (2008). IGF-II/mannose-6-phosphate receptor signaling induced cell hypertrophy and atrial natriuretic peptide/BNP expression via Gαq interaction and protein kinase C-α/CaMKII activation in H9c2 cardiomyoblast cells. J. Endocrinol..

[20] Motyka B., Korbutt G., Pinkoski M.J., Heibein J.A., Caputo A., Hobman M., Barry M., Shostak I., Sawchuk T., Holmes C.F. (2000). Mannose 6-phosphate/insulin-like growth factor II receptor is a death receptor for granzyme B during cytotoxic T cell-induced apoptosis. Cell.

[21] Chu C.H., Tzang B.S., Chen L.M., Liu C.J., Tsai F.J., Tsai C.H., Lin J.A., Kuo W.W., Bau D.T., Yao C.H. (2009). Activation of insulin-like growth factor II receptor induces mitochondrial-dependent apoptosis through Gαq and downstream calcineurin signaling in myocardial cells. Endocrinology.

[22] Chu C.H., Huang C.Y., Lu M.C., Lin J.A., Tsai F.J., Tsai C.H., Chu C.Y., Kuo W.H., Chen L.M., Chen L.Y. (2009). Enhancement of AG1024-induced H9c2 cardiomyoblast cell apoptosis via the interaction of IGF2R with Gα proteins and its downstream PKA and PLC-β modulators by IGF-II. Chin. J. Physiol..

[23] O’Reilly S.M., Leonard M.O., Kieran N., Comerford K.M., Cummins E., Pouliot M., Lee S.B., Taylor C.T. (2006). Hypoxia induces epithelial amphiregulin gene expression in a CREB-dependent manner. Am. J. Physiol. Cell Physiol..

[24] Crawford R.M., Jovanovic S., Budas G.R., Davies A.M., Lad H., Wenger R.H., Robertson K.A., Roy D.J., Ranki H.J., Jovanovic A. (2003). Chronic mild hypoxia protects heart-derived H9c2 cells against acute hypoxia/reoxygenation by regulating expression of the SUR2A subunit of the ATP-sensitive K^+^ channel. J. Biol. Chem..

[25] Abdul K.S.M., Joyanovic S., Sukhodub A., Du Q.Y., Jovanovic A. (2014). Upregulation of cardioprotective SUR2A by sub-hypoxic drop in oxygen. BBA Mol. Cell Res..

[26] Shameem K., Abdul M., Jovanovic S., Du Q.Y., Sukhodub A., Jovanovic A. (2015). A link between ATP and SUR2A: A novel mechanism explaining cardioprotection at high altitude. Int. J. Cardiol..

[27] Abdul K.S.M., Jovanovic S., Du Q., Sukhodub A., Jovanovic A. (2015). Mild hypoxia *in vivo* regulates cardioprotective SUR2A: A role for Akt and LDH. BBA Mol. Basis Dis..

[28] Lee S.D., Kuo W.W., Wu C.H., Lin Y.M., Lin J.A., Lu M.C., Yang A.L., Liu J.Y., Wang S.G.P., Liu C.J. (2006). Effects of short- and long-term hypobaric hypoxia on Bcl2 family in rat heart. Int. J. Cardiol..

[29] Chang R.L., Lin J.W., Hsieh D.J., Yeh Y.L., Shen C.Y., Day C.H., Ho T.J., Viswanadha V.P., Kuo W.W., Huang C.Y. (2015). Long-term hypoxia exposure enhanced IGFBP-3 protein synthesis and secretion resulting in cell apoptosis in H9c2 myocardial cells. Growth Factors.

[30] Chen L.M., Kuo W.W., Yang J.J., Wang S.G.P., Yeh Y.L., Tsai F.J., Ho Y.J., Chang M.H., Huang C.Y., Lee S.D. (2007). Eccentric cardiac hypertrophy was induced by long-term intermittent hypoxia in rats. Exp. Physiol..

[31] Kotla S., Singh N.K., Heckle M.R., Tigyi G.J., Rao G.N. (2013). The transcription factor CREB enhances interleukin-17A production and inflammation in a mouse model of atherosclerosis. Sci. Signal..

[32] Schauer I.E., Knaub L.A., Lloyd M., Watson P.A., Gliwa C., Lewis K.E., Chait A., Klemm D.J., Gunter J.M., Bouchard R. (2010). CREB downregulation in vascular disease: A common response to cardiovascular risk. Arterioscler. Thromb. Vasc. Biol..

[33] Ozgen N., Guo J., Gertsberg Z., Danilo P., Rosen M.R., Steinberg S.F. (2009). Reactive oxygen species decrease cAMP response element binding protein expression in cardiomyocytes via a protein kinase D1-dependent mechanism that does not require Ser133 phosphorylation. Mol. Pharmacol..

[34] Fiol C.J., Williams J.S., Chou C.H., Wang Q.M., Roach P.J., Andrisani O.M. (1994). A secondary phosphorylation of CREB341 at Ser129 is required for the cAMP-mediated control of gene expression. A role for glycogen synthase kinase-3 in the control of gene expression. J. Biol. Chem..

[35] Gonzalez G.A., Montminy M.R. (1989). Cyclic AMP stimulates somatostatin gene transcription by phosphorylation of CREB at serine 133. Cell.

[36] Balogh A., Nemeth M., Koloszar I., Marko L., Przybyl L., Jinno K., Szigeti C., Heffer M., Gebhardt M., Szeberenyi J. (2014). Overexpression of CREB protein protects from tunicamycin-induced apoptosis in various rat cell types. Apoptosis Int. J. Program. Cell Death.

[37] Bonni A., Brunet A., West A.E., Datta S.R., Takasu M.A., Greenberg M.E. (1999). Cell survival promoted by the Ras-MAPK signaling pathway by transcription-dependent and -independent mechanisms. Science.

[38] Walton M., Woodgate A.M., Muravlev A., Xu R., During M.J., Dragunow M. (1999). CREB phosphorylation promotes nerve cell survival. J. Neurochem..

[39] Ghosh P., Dahms N.M., Kornfeld S. (2003). Mannose 6-phosphate receptors: New twists in the tale. Nat. Rev. Mol. Cell Biol..

[40] Bohnsack R.N., Patel M., Olson L.J., Twining S.S., Dahms N.M. (2010). Residues essential for plasminogen binding by the cation-independent mannose 6-phosphate receptor. Biochemistry.

[41] Kuo W.W., Liu C.J., Chen L.M., Wu C.H., Chu C.H., Liu J.Y., Lu M.C., Lin J.A., Lee S.D., Huang C.Y. (2006). Cardiomyoblast apoptosis induced by insulin-like growth factor (IGF)-I resistance is IGF-II dependent and synergistically enhanced by angiotensin II. Apoptosis Int. J. Program. Cell Death.

[42] Lee S.D., Chu C.H., Huang E.J., Lu M.C., Liu J.Y., Liu C.J., Hsu H.H., Lin J.A., Kuo W.W., Huang C.Y. (2006). Roles of insulin-like growth factor II in cardiomyoblast apoptosis and in hypertensive rat heart with abdominal aorta ligation. Am. J. Physiol. Endocrinol. Metab..

[43] Montminy M. (1997). Transcriptional regulation by cyclic AMP. Annu. Rev. Biochem..

[44] MacGillavry H.D., Stam F.J., Sassen M.M., Kegel L., Hendriks W.T., Verhaagen J., Smit A.B., van Kesteren R.E. (2009). NFIL3 and cAMP response element-binding protein form a transcriptional feedforward loop that controls neuronal regeneration-associated gene expression. J. Neurosci..

[45] Matus M., Lewin G., Stumpel F., Buchwalow I.B., Schneider M.D., Schutz G., Schmitz W., Muller F.U. (2007). Cardiomyocyte-specific inactivation of transcription factor CREB in mice. FASEB J..

[46] Lin K.H., Kuo C.H., Kuo W.W., Ho T.J., Pai P., Chen W.K., Pan L.F., Wang C.C., Padma V.V., Huang C.Y. (2015). NFIL3 suppresses hypoxia-induced apoptotic cell death by targeting the insulin-like growth factor 2 receptor. J. Cell. Biochem..

[47] Li F., Liu J., Jo M., Curry T.E. (2011). A role for nuclear factor interleukin-3 (NFIL3), a critical transcriptional repressor, in down-regulation of periovulatory gene expression. Mol. Endocrinol..

